# Characterization of Crocetin Isomers in Serum Samples via UHPLC-DAD-MS/MS and NMR after Saffron Extract (Safr’Inside™) Consumption

**DOI:** 10.3390/metabo14040190

**Published:** 2024-03-28

**Authors:** Adeline Vignault, Carole Vaysse, Karène Bertand, Stéphanie Krisa, Arnaud Courtois, Benjamin Moras, Tristan Richard, David Gaudout, Line Pourtau

**Affiliations:** 1 Activ’Inside, 33750 Beychac et Caillau, France; b.moras@activinside.com (B.M.); d.gaudout@activinside.com (D.G.); l.pourtau@activinside.com (L.P.); 2 Nutrition-Health & Lipid Biochemistry Department, ITERG, 33610 Canejan, France; c.vaysse@iterg.com (C.V.); k.bertrand@iterg.com (K.B.); 3 Bordeaux INP, INRAE, OENO, UMR 1366, ISVV, University of Bordeaux, 33140 Villenave d’Ornon, France; stephanie.krisa@u-bordeaux.fr (S.K.); arnaud.courtois@u-bordeaux.fr (A.C.); tristan.richard@u-bordeaux.fr (T.R.)

**Keywords:** saffron extract, crocin, crocetin, isomers, metabolism, UHPLC-DAD-MS/MS, NMR

## Abstract

The therapeutic effects of saffron have been reported and described in relation to its major derivatives. Among them, in terms of saffron’s properties, crocin and crocetin absorption and bioavailability have been the most studied. Nevertheless, the metabolism of these major compounds of saffron has not yet been entirely elucidated. Current data indicate that the phase 2 metabolism of crocetins go through conjugation reactions. Crocetins could also be present in isomeric forms such as other carotenoids. Nonetheless, there are still shadow areas in regard to the measurements of the different circulating forms of crocetins after oral saffron extract administration (Safr’Inside™). In using various approaches, we propose the identification of a new *cis* isomeric form of crocetin, the 6-*cis*-crocetin. This compound was found in human serum samples after an oral administration of saffron extract. The 6-*cis*-crocetin represents 19% of the total crocetin measured after 45 min of consumption. These data mark, for the first time, the presence of a *cis* isomeric form of crocetin in human serum samples. Moreover, this study led to the development of an analytical method that is able to identify and quantify both isomeric forms (*trans* and *cis*).

## 1. Introduction

Saffron is a spice used as flavoring and coloring in food, produced from the dried stigmas of Crocus sativus flowers. Saffron is also known for a wide range of health-promoting benefits in traditional and modern medicine [1]. Numerous scientific studies have highlighted the positive biomedical and pharmacological properties of saffron, specifically in regard to the nervous system, cardiovascular system, immune system and even cancer [2,3]. However, despite this growing number of pre-clinical and clinical studies, there is still a great deal of uncertainty surrounding the natures of molecules derived from saffron that exert bioactivity. Indeed, saffron stigmas contain more than 150 volatile and coloring compounds, but only a few of them have been completely characterized regarding bioactivity.

The four main compounds that have been most widely described for their contribution to saffron properties include safranal, picrocrocine, crocin and crocetin. Safranal gives saffron its distinctive aroma, while picrocrocin contributes to its bitter taste and flavor. Crocins and crocetins are saffron carotenoid pigments, giving pure saffron its red color. More precisely, crocetin, and its ester form (crocin), are derived from other carotenoids (a-carotene and b-carotene, lycopene and zeaxanthin) present in saffron stigma. They can be present in both their *trans* or *cis* isomeric forms and result from the plant metabolism of zeaxanthin, which is itself produced from b-carotene. Crocetins esterified with sugars (such as glucose, gentobiose or neapolitanose) produce the geometric isomers of crocins, where *trans* isomers dominate over their *cis* forms [4].

The potential therapeutic activity of saffron has mainly been studied in relation to its major derivatives such as crocin and crocetin, of which their bioactivity may be due to their anti-oxidative and anti-inflammatory properties [5]. Safranal has also been studied as a promising therapeutic agent for various CNS functions (anxiety, memory enhancement) and diseases such as Alzheimer’s, Parkinson and Huntington disease [6]. The bioavailability of saffron molecules is currently poorly described, and studies have mainly focused on the crocin or crocetin compounds, where it has been shown that crocins and crocetins have poor absorption and low bioavailability [1].

**Scheme 1:**
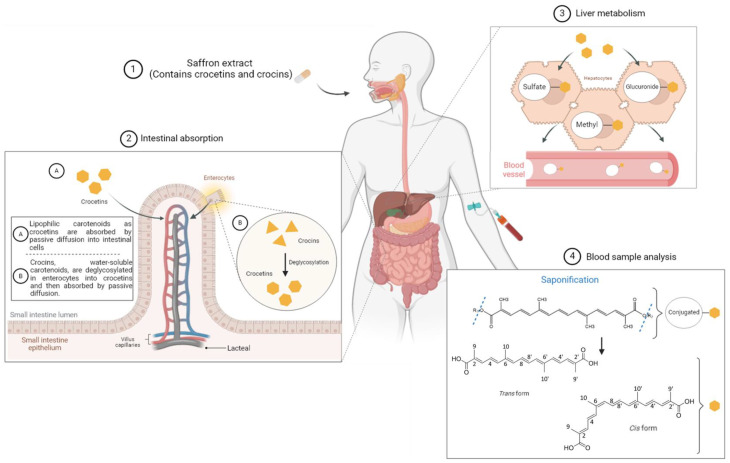
Absorption and metabolism after the oral administration of a saffron extract.

After the oral administration of saffron extract, crocins are rapidly hydrolyzed to deglycosylated crocetin, mainly by enzymes of the intestinal epithelium, which are absorbed through passive diffusion through the intestinal barrier [7] (Scheme 1).

Thus, it seems that only *trans*-crocetin can reach the bloodstream and undergo phase 2 metabolism, resulting in conjugated metabolites (glucuronides, sulfates, methylates, etc.). This raises the question of how to evaluate the bioavailability of crocetins, since they are present in several conjugated metabolite forms. Various methods of sample preparation from clinical and pre-clinical studies have been described in the literature [8,9,10,11,12,13,14,15], including a saponification step. Indeed, saponification should release the conjugated forms as already for the carotenoid analysis in serum [16]. This step will help to liberate the crocetins from the conjugated group and quantify all the circulating forms of crocetins (Scheme 1).

Like other carotenoids, *trans*-crocetin can isomerize during the digestion of crocin. The isomers produced could have a significant biological effect and therefore deserve to be quantified. Indeed, lycopene metabolism (precursor of *trans*-crocetin in the plant metabolism) has been shown to isomerize from the *trans* to various *cis* forms in vitro [17].

Furthermore, it has been demonstrated that *cis*-lycopene isomers have a higher antioxidant capacity than *trans* forms in vitro [18]. To our knowledge, no method has yet investigated the analysis of crocetin isomeric forms in human samples. It is therefore essential to develop a method enabling the detection and quantification of crocin’s metabolite isomers (*cis*/*trans*), with biological potential circulating in the bloodstream.

The primary aim of this work was built on a two-step approach. The first step consisted of exploring the possibility of inducing the physicochemical isomerization of *trans*-crocetin into *cis*-crocetin in order to characterize and use it as a reference molecule. In a second step, *cis*-crocetin forms were analyzed in biological samples from clinical studies, validating the hypothesis regarding the formation of this/these isomer(s) in vivo. The second aim was to validate the analytical method for the determination and quantification of these isomers.

## 2. Materials and Methods

### 2.1. Material and Reagents

Crocin (also named crocin-I, molecular weight: 976.96, purity: 95%) and crocetin (molecular weight: 328.4, purity: 95%) were purchased from Techlab (Metz, France). Acetonitrile, water, methanol and formic acid were of LC-MS grade and purchased from Fisher Scientific (Pittsburgh, PA, USA). Ethyl acetate, dimethyl sulfoxide (DMSO) and sterile-filtered serum (hereafter referred to as “blank standard serum” free of crocin and crocetin; ref H4522) were obtained from Sigma-Aldrich (St. Louis, MO, USA). Potassium hydroxide (KOH) was obtained from VWR chemicals (Pessac, France). DMSO-d6 was purchased from Euriso-Top (Cambridge Isotop Laboratories, Inc., Saint-Aubin, France).

### 2.2. Identification of Trans-Geometrical Isomers of Crocetins

#### 2.2.1. Isomerization of Trans-Crocetin through Heat Treatment

A solution of *trans*-crocetin of 2 mg/mL was prepared in DMSO-d6 in order to be analyzed via UHPLC-DAD-MS/MS, as well as via NMR, to identify the structure of the produced compound. The freshly prepared *trans*-crocetin solution was divided in two after shaking and vortexing into an NMR tube containing 750 µL of the solution and into a UHPLC vial containing 250 µL. Directly after the distribution, the UHPLC vial and the NMR tube were then placed simultaneously in a water bath at 100 °C for 1 h every day for 7 days, without protection from light. Between the heating times, the samples were kept in an oven at 30 °C, without any protection from light. The analyses of the UHPLC vial and the NMR tube were performed at D+3, D+4 and D+7 in order to follow the isomerization of *trans*-crocetin. All experiments were performed in triplicate.

#### 2.2.2. UHPLC-DAD-MS/MS Characterization of Geometrical Trans-Crocetin Isomer

The samples prepared as described in Section 2.2.1 were diluted 1:1000 (*v*/*v*) with DMSO-d6 and directly injected into a UHPLC-DAD-MS system. The UHPLC was a Vanquish™ high-performance liquid chromatography system coupled to a diode array detector (DAD) and a TSQ-Fortis mass spectrometer detector (Thermo Fisher Scientific Inc., Waltham, Massachusetts, USA). The control software was FreeStyle™ 1.7 (version 1.7.73.12). The TSQ-Fortis used a heated electrospray ionization (H-ESI) source operated in the negative ionization mode, and the following parameters were set: capillary voltage, 2500 V; sheath gas, 50 psi; aux gas, 10 psi; sweep gas, 1 psi; ion transfer tube temperature, 300 °C; and vaporizer temperature, 350 °C. Samples were analyzed via injection (1 µL) on an Atlantis T3 column (100 Å, 2.1 × 100 mm, 3 µm particle size; Waters, France). The solvents, at a flow rate of 0.2 mL/min, were water acidified with 0.1% of formic acid (solvent A) and acetonitrile (solvent B). The elution gradient was (time, % of solvent A) 0 min, 90.0%; 2 min, 90.0%; 5 min, 50.0%; 8 min, 50.0%; 11 min, 25.0%; 14 min, 25.0%; 20 min, 0.0%; 25 min, 0.0%; and 30 min, 90.0%, and then, 6 min of equilibrium time was left between each analysis. Compounds were identified using the DAD running from 195 to 700 nm, the visible spectra at 440 nm, the “SRM mode” that allowed the input of the exact mass of the searched compounds, the fragments associated with specific collision energy and tube lens. The SRM parameters in negative mode were the following: *m*/*z* = 327.16; product ion 1, *m*/*z* = 239.14; product ion 2, *m*/*z* = 283.05; collision energy 1, 11 eV; collision energy 2, 8 eV; and tube lens, 71 V.

#### 2.2.3. NMR Structural Identification of Geometrical Trans-Crocetin Isomer

Crocetin isomerization was monitored in situ on a Bruker Avance III 600 MHz (Bruker, Wissembourg, France) spectrometer using 1D- and 2D-NMR experiments acquired at 293 K. The NMR was operating at 600.27 MHz and equipped with a TXI 5 mm probe with z gradient coils. The measurements were performed using Topspin 4.0.8 software (Bruker, Wissembourg, France).

### 2.3. Identification of Trans-Geometrical Isomers of Crocetin after Oral Administration of Saffron Extract (Safr’Inside™) in Human Samples

Thanks to the experiments conducted in the previous part (Section 2.2), we were able to form 6-*cis*-crocetin and determine its characteristics (retention time, maximum absorption wavelength and MS parameters), which helped identify its presence in human biological samples. Prior to this, it was ensured that the saponification step in the biological sample preparation did not affect the natural in vivo isomerization of *trans*-crocetin.

Firstly, to identify and quantify *trans*-crocetin isomers, it was important to elucidate their presence in human biological samples. With this aim, a saffron extract (Safr’Inside™; patent FR 3054443-WO2017EP69200) provided by Activ’inside (Beychac et Caillau, France) containing crocins > 3% (divided into 83.0% *trans*-crocins and 17.0% *cis*-crocins) and safranal > 0.2% (measured by UHPLC-DAD) was administered to the volunteers of the clinical study in order to monitor metabolite formation.

#### 2.3.1. Clinical Study

This study was conducted in accordance with the Declaration of Helsinki of 1975 (https://www.wma.net/what-we-do/medical-ethics/declaration-of-helsinki/, accessed, on 1 April 2021), revised in 2013. The human study was approved by the French Ethical Committee (2021T2-02 RIPH2 HPS/N_ SI RIPH: 21.01.11.58647/N_ EudraCT/ID RCB:2020-A3184-35/Comité de Protection des Personnes CPP Tours-Région Centre-Ouest I; approved 11 March 2021). The volunteers provided their written informed consent before they participated in the study.

A total of 10 healthy male volunteers (age: 25.0 years old, +/−5.1; BMI: 23.9 kg/m^2^, +/−2.3; >60 kg; without drug treatment and no distinction with regard to ethnicity) with normal biochemical parameters were enrolled in this study. These subjects were administered a single oral dose of a 300 mg saffron extract (Safr’Inside™) capsule. Blood samples were collected at 0, 5, 10, 15, 30, 45, 90, 120, 150, 180, 210, 240 and 300 min after saffron administration. Blood samples were collected in serum-separating tubes, prepared and immediately frozen until analysis [19].

#### 2.3.2. Analysis of Samples

A total of 200 µL of acetonitrile, causing deproteinization, and 20 µL of formic acid were added to 200 µL of human serum in an Eppendorf tube. After centrifugation at 10,000× *g* for 10 min at 4 °C, the supernatant was transferred in a 10 mL glass tube. The precipitant was resuspended in 200 µL of water, 200 µL of acetonitrile and 20 µL of formic acid. This step was repeated twice, and the three supernatants were pooled and saponified with 2 mL of a solution of 6% KOH in water (*w*/*v*) for 5 min at 100 °C in a water bath (with a vortex agitation each minute). After cooling in ice, 4 mL of an aqueous solution of formic acid 7% (*v*/*v*) was added to the sample. Crocetin was then extracted three times with ethyl acetate (3 mL) followed by vortexing for 5 min and a centrifugation at 1900× *g* for 5 min at 4 °C. The three successive supernatants were pooled and evaporated under nitrogen. The final dry residue was dissolved in 500 µL of methanol/water (80/20) and transferred in a glass amber vial with inserts. Samples were analyzed using the UHPLC-DAD-MS/MS conditions described in Section 2.2.2. The results were expressed as the percentage of each compound formed (regarding total crocetin, i.e., both isomers) since not all standards were available.

### 2.4. Method Validation

#### 2.4.1. Standard Solutions and Quality Control Serum

A stock solution of standard crocetin was first prepared in DMSO (1 mg/mL) and then diluted in methanol/water (80/20) to reach a concentration of 10 µg/mL. Dilutions were prepared from 1 to 15 ng/mL and stored in glass amber vials at −80 °C until analysis. All standards were processed and manipulated under dim light to protect against light-induced isomerization or possible degradation.

The stock solution of standard crocin used to spike the reference serum was prepared by first mixing crocin in water and then dissolving it in methanol to a final volume of 10 mL. This stock solution was then gradually diluted in methanol/water (80/20) into a series of concentrations as a working solution. All the working solutions were stored in glass amber vials at −80 °C, before use.

The Quality Control (QC) serum samples were prepared at three concentrations (11 ng/mL, 55 ng/mL, 110 ng/mL) by spiking blank standard serum sample with known amounts of standard crocin. These spiked blank standard serum samples were vortexed under dim light, for 30 min, and then dispensed in 2 mL aliquots in Eppendorfs and stored at −80 °C until analysis. These spiked standard serum samples were analyzed according to the protocol described in Section 2.4.2.

#### 2.4.2. UHPLC-MS/MS Method Development

- Instrumentation

UHPLC-MS/MS was carried out on a Thermo Scientific™ Vanquish™ ultra-high-performance liquid chromatography (UHPLC) system (Thermo Fisher scientific, Waltham, MA, USA), consisting of a quaternary pump and an autosampler coupled to a TSQ Altis triple quadrupole mass spectrometer (Thermo Fisher scientific, Waltham, MA, USA) equipped with a heated electrospray ionization (H-ESI) source. Chromatographic separation was performed on a reversed-phase column Atlantis T3 C18 (100 × 2.1 mm; 3 µm) column (Waters, Milford, CT, USA), maintained at 25 °C.

- Chromatographic conditions

For chromatographic separation, the following combination of mobile phases were used. Mobile phase A consisted of 0.1% formic acid in water, and mobile phase B, acetonitrile. The following linear gradient of B was used (t (min), %B): (0–2, 10%); (2–5, 10–50%); (5–8, 50%); (8–11, 50–75%); (11–14, 75%); (14–20, 75–100%); (20–−5, 100%); (25–30, 100–10%); and (30–36, 10%). The total run time of analysis was 36 min. The mobile phase flow rate was 0.2 mL/min, and 20 µL of the sample was injected into the UHPLC system.

- MS Conditions

A triple quadrupole mass spectrometer TSQ Altis equipped with a heated electrospray ionization (H-ESI) source and controlled using Chromeleon Analyst software (version-7.2.10) (Thermo Fisher scientific, Waltham, MA, USA) was used for direct infusion experiments. The standard of crocetin was pre-screened to select the respective mass of its deprotonated precursor and its product ions. The spray voltage was set to 3500 V in negative mode. The ion transfer tube temperature was 350 °C, and the vaporizer temperature was 350 °C. Nitrogen gas was used as a sheath and as an auxiliary gas at a flow rate of 50 arbitrary units (arb) and 15 arb, respectively. The chosen multiple reaction monitoring (MRM) transitions for the crocetin were 327.1 (*m*/*z*) for the precursor and 239.2 (*m*/*z*) for the product ions. The optimal values of the RF lens and collision energy (CE) for crocetin were, respectively, 131 V and 12 eV.

#### 2.4.3. Method Validation

The present method was validated according to the criteria of the ICH guidelines [20].

The quality parameters established for the validation of the method were linearity; precision and accuracy; limit of detection (LOD) and limit of quantification (LOQ); recovery and matrix effect; and stability.

- Linearity

Standard serial dilutions of crocetin (1 à 15 ng/mL) were run on UHPLC-MS/MS. The peak area of detected product ions was plotted against their concentration. Linearity was expressed as the linear coefficient (r2) with a value that was expected to be >0.99.

- Precision and accuracy

The intra-day accuracy and precision were determined by analyzing three replicates of QC serum samples spiked at three levels of crocin (11 ng/mL, 55 ng/mL, 110 ng/mL, after saponification and corresponding to an equivalent crocetin concentration of 3 ng/mL, 16 ng/mL, 29 ng/mL).

The intra-day precision and accuracy were examined in one day at each of the three concentration levels. For the inter-day analysis, the same spiked QC serum sample set was analyzed on four consecutive days.

The precision of the developed method was evaluated using the %RSD (percentage of relative standard deviation of intra- and inter-day repeatability). The values determined at each concentration level should not exceed 15% of the RSD.

The accuracy was expressed as the percentage of the relative error values of the difference between the mean observed concentration and the known spiked concentration in the plasma matrix. The mean accuracy should be within 15% of the nominal value.

- Limits of detection and quantification

The limit of detection (LOD) and limit of quantification (LOQ) of the method were determined from the chromatograms of spiked blank plasma at the lowest crocin concentration tested for a signal-to-noise ratio of 3. The LOQ was determined for a signal-to-noise ratio of 10.

- Recovery and matrix effect

The recovery of crocetin was calculated from the 3 sets of spiked QC serum samples. Following extraction, recovery (%) was determined by comparing the mean concentration of crocetin measured in the QC serum samples with the initial amount of equivalent crocetin added in the blank standard serum.

The matrix effect of crocetin was performed at low and medium concentrations, in three replicates. The matrix effect was assessed through a comparison of the peak areas of crocetin spiked in extracted blank serum with those of the standard solution at corresponding concentrations, and the ratio of peak areas was regarded as the matrix effect.

- Stability

The stability of the crocetin in human serum was determined by measuring 3 replicates of serum samples at QC levels, under different conditions: after storage at room temperature for 4 h, after storage at 4 °C for 24 h, after three freeze–thaw cycles and 30 days stocked at −20 °C to room temperature. The stability results should be within 15% of the nominal concentrations.

## 3. Results

### 3.1. Identification of Trans-Geometrical Isomers of Crocetin Standard

#### 3.1.1. Detection of Trans-Geometrical Isomers of Crocetin via UHPLC-DAD/MS after Heat Treatment

To induce the isomerization of *trans*-crocetin into *cis*-crocetin in situ, drastic conditions were applied, i.e., 1 h/day heating at 100 °C for several days unprotected from light. The samples were analyzed over time in order to follow the evolution of the *trans*-crocetin form via UHPLC-DAD-MS/MS using the SRM mode.

**Figure 1:**
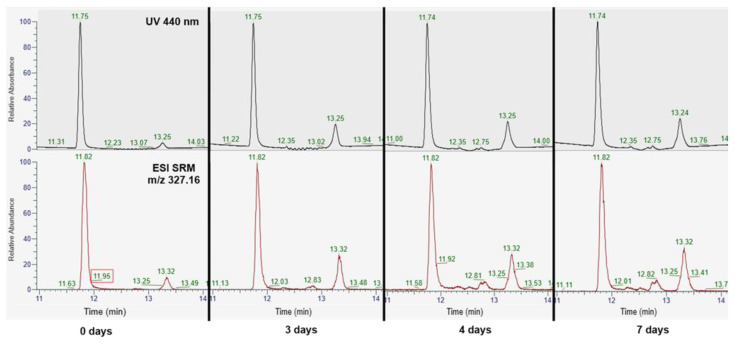
UV 440 nm chromatograms and ESI SRM *m*/*z* 327.16 spectra at days 0, 3, 4 and 7 of *trans*-crocetin isomerization.

Figure 1 shows UV-Vis chromatograms at 440 nm and ESI SRM spectra of the parent ion, *m*/*z* = 327.16 from Day 0 to Day 7 after *trans*-crocetin isomerization. At D+0, a major peak was observed at 11.75 min, and a very minor peak was observed at 13.25 min in the UV-Vis and MS. The major peak at 11.75 min corresponds to the *trans*-crocetin standard used for the experiment, but the standard was not 100% pure, explaining the presence of a second peak.

As the kinetics progress, the peak observed at 13.25 min grew more and more in intensity. This peak is visible at 440 nm and responds in mass to the parent ion *m*/*z* = 327.16 as does *trans*-crocetin (Table 1). In order to better identify the compound formed at 13.25 min, the DAD spectrum of this compound was recorded between 200 and 700 nm, as well as that of *trans*-crocetin for comparison (Table 1).

**Figure 2:**
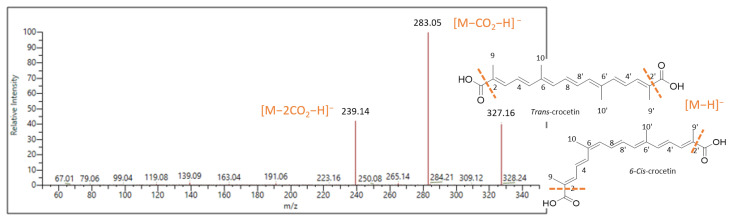
Fragmentation patterns of *trans*- and 6-*cis*-crocetin. The cleavage of 1 dotted line on the molecules corresponds to a decarboxylation-giving ion *m*/*z* 283.05, and the cleavage of 2 dotted lines on the molecules corresponds to a di-decarboxylation-giving ion *m*/*z* 239.14.

Regarding the peak at 11.75 min, two maximum absorptions were observed at 255 nm and 440 nm, respectively (Table 1). Regarding the peak at 13.25 min, the same two absorption maxima were observed, except that this time, a third absorption maximum was noted at 320 nm (Table 1). In addition, both compounds presented the same fragmentation and product ions corresponding to mono- and di-decarboxylation as shown in Figure 2.

#### 3.1.2. Characterization of Trans-Crocetin Isomer via NMR after UHPLC-DAD/MS Identification

**Figure 3:**
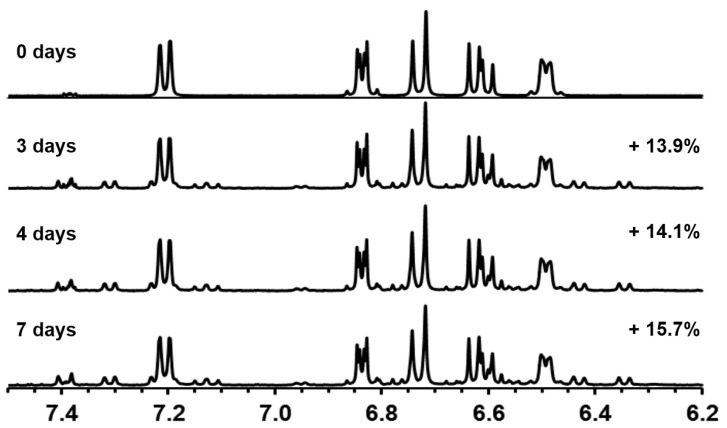
In situ isomerization of *trans*-crocetin monitored through ^1^H-NMR experiments: ^1^H-NMR spectrum after 7 days of heating.

The *trans*/*cis*-isomerization of crocetin was also monitored through in situ NMR experiments. Isomerization was induced through the external heating of the NMR tube 1 h/day at 100 °C for seven days, as previously performed for the UHPLC-DAD-MS/MS analysis. ^1^H-NMR spectra were acquired every day as shown in Figure 3.

On day 0, the ratio *trans*/*cis*-isomers from the ^1^H-NMR spectrum indicates the absence of the *cis*-crocetin form. On the contrary, after only 3 days of heating, almost 13.9% of the *cis*-crocetins were formed.

**Figure 4:**
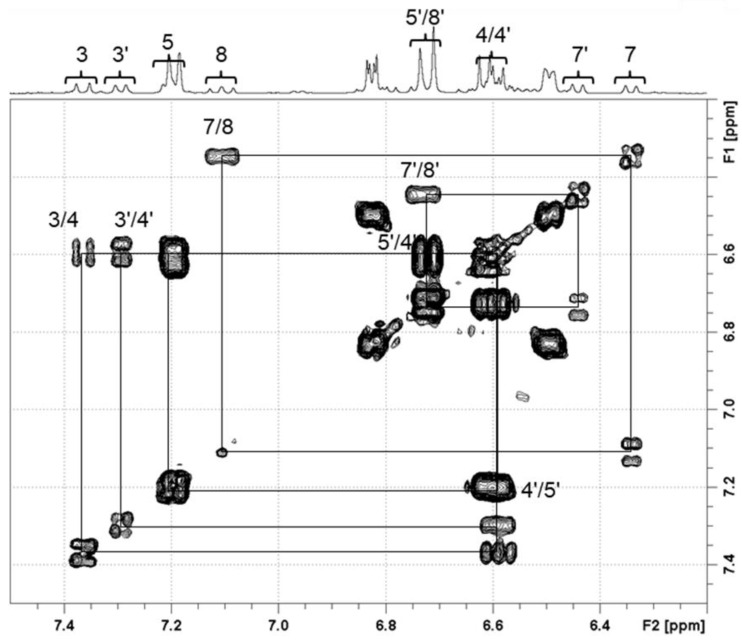
In situ isomerization of *trans*-crocetin monitored through ^1^H-NMR experiments: 2D-COSY spectrum after 7 days of heating.

On day 4, no important change was detected compared to day 3: only a +0.2% increase in the *cis*-crocetin form was observed. The isomerization was a little bit more important between days 4 and 7 with 1.6% of *cis*-crocetin production. After 7 days of treatment, 15.7% of the *trans*-crocetin was isomerized in *cis*-crocetin under the heating condition. This production of *cis*-crocetin is sufficient to characterize the form with NMR using a ^1^H 2D COSY spectra and to therefore determine where the isomerization will take place on the carbon chain. For this purpose, 2D NMR experiments were performed at the beginning and at the end of the isomerization process. These results confirm the formation of a *cis*-isomer. The structure of the *cis*-isomer was identified through a comparison with the literature [21] based on proton and 2D-COSY spectra, as presented in Figure 4.

The ^1^H-NMR data of *trans*- and *cis*-isomers of crocetin are reported in Table 2. Differences between the two isomers appear for the olefinic protons due to the loss of symmetry. The ^1^H-NMR spectrum of *trans*-crocetin contains five resonances in the olefinic region. The first set of resonances is constituted of a doublet of doublets at 6.61 ppm (dd, 11.4 and 15.0 Hz) corresponding to protons 4/4′.

**Figure 5:**
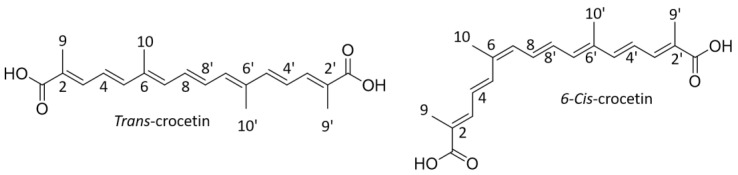
Structure of *trans*- and 6-*cis*-crocetin forms.

These protons were coupled to protons 3/3′ at 7.21 ppm (d, 11.4 Hz) and protons 5/5′ at 6.73 ppm (d, 15.0 Hz). A second set of resonances is constituted by the signal at 6.49 ppm (dd, 3.0 and 7.9 Hz), attributed to protons 7/7′, which are coupled with protons 8 and 8′ at 6.84 ppm (dd, 3.0 and 7.9 Hz). Concerning the *cis*-crocetin spectra, the main chemical shift differences appear for protons 3, 5, 7 and 8, in comparison with that of the *trans*-crocetin. Protons 3, 5 and 8 are shifted downfield by 0.18, 0.46 and 0.26 ppm, respectively, whereas proton 7 is shifted upfield by 0.16 ppm. These differences and the coupling constants measured between these protons are in agreement with those observed for the isomerization of the crocetin digentiobiosyl ester [21]. These results are consistent with the formation of the 6-*cis*-crocetin after heating (Figure 5).

### 3.2. Identification of Trans-Geometrical Isomers of Crocetin after Oral Administration of Saffron Extract (Safr’Inside™) in Human Samples

**Figure 6:**
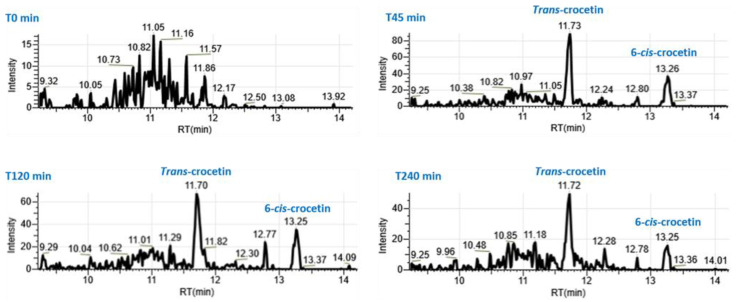
SRM chromatograms of patient at T0 and over time (45, 120 and 240 min) showing the appearance of 6-*cis*-crocetin.

Crocetins were monitored from time 0 to 300 min after the oral administration of Safr’Inside™, followed by the collection of human serum samples. As an example, Figure 6 describes one volunteer’s SRM chromatograms at different times (0, 45, 120 and 240 min) after the oral administration of saffron extract (Safr’Inside™). Two metabolites resulting from the ingestion of Safr’Inside™ could be highlighted and characterized using the new method, namely the *trans*-crocetin (retention time: 11.70 min) and the 6-*cis*-crocetin (retention time: 13.26 min).

**Figure 7:**
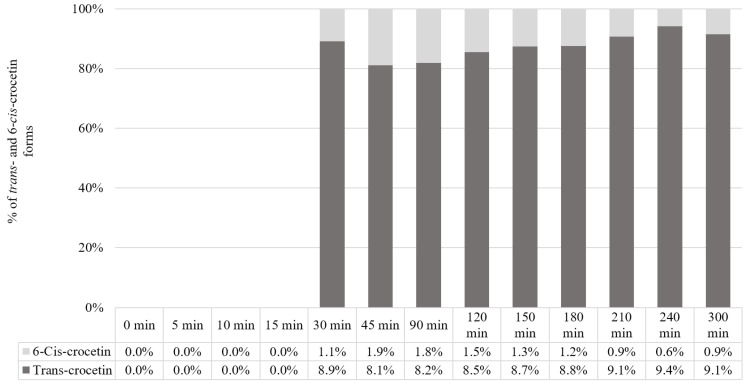
Mean of isomeric percentage of *trans*-crocetin vs. 6-*cis*-crocetin after oral administration of saffron extract in human samples during time analyzed via UHPLC-DAD/MS.

After showing that both isomers were detected in the biological samples, it was of interest to determine their respective proportions. Figure 7 shows the average percentage of both compounds found in the 10 human sera at the various times after ingestion of the extract. It can be seen that at up to 15 min after the administration of the extract, neither of the two metabolites are found in the serum. Nevertheless, from 30 min, we observed the appearance of these two forms until the end of the kinetics (i.e., at 300 min after the ingestion of the extract). Likewise, we can see that the percentage of induced 6-*cis*-crocetin increased from 11% to 19% between 30 and 90 min before decreasing in favor of the *trans*-crocetin form, which increased from 85.6% to 91.4% between 120 and 300 min. In a global way, we observed a very majority presence of the *trans*-crocetin form with an average of 80–90% against only 10 to 20% for the 6-*cis*-crocetin form.

### 3.3. Method Validation

**Figure 8:**
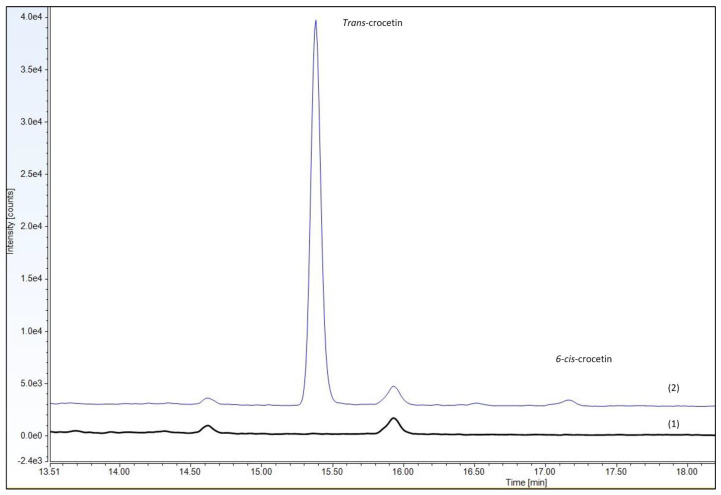
Typical MRM chromatograms of (1) blank serum and (2) blank serum sample spiked with standard.

The method’s quality parameters were studied under optimum separation parameter conditions using human serum spiked with crocin. Validation was carried out as per USFDA and ICH-Q2 guidelines [20]. The representative multiple reaction monitoring (MRM) chromatograms of crocetin in human serum are shown in Figure 8. Compared with the chromatogram of blank serum sample, there were no endogenous interference peaks at the retention times of *trans*-crocetin and 6-*cis*-crocetin in the spiked serum samples.

All the others validation parameters are presented in Table 3. The calibration curves of *trans*-crocetin showed linear responses ranging from 0.9 to 150 ng/mL in serum. The limit of detection was found to be 0.09 ng/mL, and the limit of quantification was found to be 0.30 ng/mL. Therefore, this method was considered sensitive for the quantification of crocin metabolites in human serum. The intra-day precision (RSD %) of the crocetin was less than 5.0%, and its accuracy (RE %) ranged from −10.0 to 13.4%. Similarly, the inter-day precision (RSD %) of crocetin was less than 6.0%, and the accuracy (RE %) ranged from −7.9 to 6.0%. The precision and accuracy of this method meet the acceptance criteria for FDA guidelines, demonstrating that the method is accurate, reliable, reproducible and acceptable.

The mean extraction recoveries of crocetin at three different concentrations ranged from 92.1 ± 2.9% to 108.1 ± 8.2% with an RSD less than 10%. The matrix effect was from 93.4 ± 2.6% to 96.6 ± 1.1% with an RSD less than 5%. The data show that the extraction recovery and matrix effect of this method were reliable and reproducible.

The results indicate that the crocetin in serum was stable at different storage conditions. The precision was found for room temperature within 4 h at 0.5% with an accuracy (RE) of −8.5%, for 24 h at 4 °C at 5.6% with an accuracy (RE) of −0.6% and at −20 °C a month at 3.6% (RE, −7.3%). After three freeze–thaw cycles (−20 °C to room temperature, freeze–thaw stability), the precision was 2.0% with an accuracy (RE) of −12.4%.

## 4. Discussion

Firstly, we physicochemically forced the isomerization of the *trans*-crocetin standard to generate a reference of the *cis* isomeric form. After a week under drastic conditions (1 h/day at 100 °C), we obtained a new compound, which could be identified and characterized via UHPLC-DAD-MS/MS and NMR. This new compound could be an isomeric form of *trans*-crocetin, since crocetin exists in *trans* and *cis* forms in the plant kingdom. The spectra confirmed the *cis*-crocetin form as it was consistent with previous studies that have shown that in the case of the *trans*- and *cis*-crocin forms, the same differences between the two UV spectra (max at 320 nm) were notable [22,23]. Moreover, the NMR characterization evidenced that this isomerization occurred at position number 6. Interestingly, for the first time, this compound was identified as 6-*cis*-crocetin.

After the identification and characterization of 6-*cis*-crocetin, it was important to determine its presence or absence in serum samples from clinical studies after an oral administration of Safr’Inside™. It was therefore necessary to have a method capable of analyzing not only *trans*-crocetin, but also 6-*cis*-crocetin. According to this, we developed and validated a method for the analyses via UHPLC-MS/MS of the geometrical isomers (*trans* and *cis*) of crocetin in human serum. In order to efficiently recover crocetin from a serum sample, pre-treatment protocols were evaluated. Generally, the methods described in the literature consist of a protein precipitation with various solvents systems (methanol, ethanol, acetonitrile), followed in some cases by a SPE purification [10,11,24] prior to analysis via UHPLC-DAD [13] or UHPLC-MS/MS [14,25,26]. Assuming that the metabolized forms of crocetins in serum are esterified crocetins under conjugated form [11] and maybe also bound to albumin [27], it can be questioned if these methods allow for the quantification of the total amount of crocetin (free, conjugated and isomeric forms). For these reasons, few methods add an enzymatic pretreatment of the serum sample with glucuronidase in order to hydrolyze the glucuronide conjugate form of crocetin. In our cases, we chose to add a saponification step after the precipitation step. As already described for the carotenoid analysis in serum [16], saponification should release the conjugated forms, allowing us to determine the total amount of crocetin in plasma. However, it was necessary to ensure that the saponification step (i.e., 5 min at 100 °C) would not be responsible of the isomerization of *trans*-crocetin in serum samples since carotenoids are known to be sensitive to light and heat [28]. Manipulations were carried out on the crocetin standard under drastic conditions in an attempt to chemically isomerize *trans*-crocetin to *cis*-crocetin to clearly demonstrate that isomerization would not be due to the analytical conditions. In fact, it took several hours over several days of heating at 100 °C to induce isomerization, far more than the 5 min at 100 °C of the proposed method. Concerning the method, we obtained very high recovery rates (>90%). Moreover, the LOD and LOQ values obtained attest to the favorable sensitivity of the method, in the ng/mL range, as well as favorable linearity, with a coefficient greater than 0.99. In addition, the accuracy and precision values are very good, although some are greater than 5%, but always less than 10%. The comparison of the stabilities of the QC samples with those of freshly prepared and instantly analyzed samples showed %RSD and %RE values within the acceptable range (below 15%). Crocetins were found to be stable enough for at least three freeze and thaw cycles and also for the investigated time periods. These results show that the samples could remain for at least 30 days at −20 °C as no out-of-limit degradation was observed.

This method was applied to samples from human clinical studies and revealed the presence of the two compounds (i.e., *trans*-crocetin and 6-*cis*-crocetin) 30 min after the oral administration. By analogy, between the sample in which the isomerization was forced and the samples from the clinical studies, we can conclude that this is the same compound and therefore affirm that we were in the presence of two isomers of crocetin (i.e., *trans*-crocetin and 6-*cis*-crocetin) after the administration of Safr’Inside™. Furthermore, the percentage of the 6-*cis*-crocetin isomer formed was greater and, above all, much faster in the case of human metabolism than when isomerization was forced physicochemically. In fact, it took 7 days to obtain 15% of the *cis*-isomeric form from the crocetin standard, compared with only 45 min after the oral administration of saffron extract to humans to obtain 19%. Crocins ingested in the extract therefore appeared to be rapidly metabolized in both isomeric forms of crocetin, with both metabolites appearing simultaneously as early as 30 min. The 6-*cis*-crocetin form is therefore formed much more quickly in vivo than in situ. However, it is important to note that the in situ isomerization was carried out starting from a crocetin standard, whereas in the case of the in vivo study, the participants ingested a saffron extract (Safr’Inside™) mainly rich in crocins. The percentage of 6-*cis*-crocetin form observed via NMR was therefore solely due to the isomerization of *trans*-crocetin itself. On the contrary, in the case of the in vivo study, the percentage of 6-*cis*-crocetin formed was possibly due to the metabolism of crocins into 6-*cis*-crocetin directly and/or through the metabolism of crocins into *trans*-crocetin, themselves then metabolized into 6-*cis*-crocetin. In the case of saffron extract, crocetins can also be absorbed in their *trans* or *cis* form, but the 6-*cis*-crocetin proportions found in human samples cannot only be explained by a potential preferential absorption of *cis*-crocetin forms. Indeed, in the case of lycopenes, for a carotenoid just like crocin and crocetin, it has been shown that the hypothesis of the preferential absorption of lycopene *cis*-isomers could not explain their increased proportion in human samples. This suggests that additional in vivo isomerization may take place [29]. Definitely, it has been shown that the key site of lycopene isomerization is inside the intestinal cells resulting in 29% of lycopene as *cis*-isomers [17]. For crocetins, the proportion of *cis*-isomers in the human clinical samples is a little lower (19% maximum at 45 min vs. 29%) than in the case of lycopene, though still remarkable.

Regarding the high proportion of *cis*-lycopene isomers in human tissues, it has been demonstrated that *cis*-lycopene isomers have a higher antioxidant capacity than *trans*-forms in vitro, suggesting that this isomerization is a metabolic activation [18]. Given that we were able to observe the same type of phenomenon in crocetins, we may well wonder whether 6-*cis*-crocetin might offer higher bioactivity than *trans*-crocetin. It will then be of interest to study the biological effects of this compound in the future.

To conclude, using a novel approach with UHPLC-DAD-MS/MS, we evidenced the formation of *cis*-crocetin after Safr’Inside™ consumption in human serum samples. Furthermore, using NMR we were able to identify the exact structure of the new compound and identified it for the first time as 6-*cis*-crocetin. This compound was then found in human serum samples after the oral administration of Safr’Inside™, representing 19% after 45 min of the total crocetins (*trans* and *cis* forms).

## 5. Patents

Safr’Inside^TM^ used in this study is a patented saffron extract (WO2018020013).

## Figures and Tables

**Table 1:** UV and MS/MS fragmentation patterns.

Compounds	Tentative Identification	Retention Time (min)	Parent Ion *m*/*z* (Negative Mode)	Product Ions *m*/*z* (Negative Mode)	Maximum Wavelength (nm)
Peak 1	*Trans*-crocetin	11.75	327.16	239.14; 283.05	255; 440
Peak 2	Unknown (Supposed 6-*cis*-crocetin)	13.25	327.16	239.14; 283.05	255; 320; 440

**Table 2:** ^1^H-NMR data of *trans*- and *cis*-isomer of crocetin.

	*trans*-Isomer		*cis*-Isomer	
Proton	δ (ppm)	*J* (Hz)	δ (ppm)	*J* (Hz)
3	7.21	d (11.4)	7.39	d (11.9)
3′			7.31	d (11.9)
4	6.61	dd (15.0, 11.4)	6.58	dd (11.9, 14.1)
4′				
5	6.73	d (15.0)	7.19	n.o.
5′			6.60	n.o.
7	6.49	dd (7.9, 3.0)	6.33	d (11.9)
7′			6.43	d (11.9)
8	6.84	dd (7.9, 3.0)	7.10	dd (11.9, 14.0)
8′			6.72	n.o.
9	1.91	s	1.92	s
9′				
10	1.97	s	1.97	s
10′				

δ: chemical shift; *J*: coupling constant; ppm: parts per million; Hz: Hertz; d: doublet; dd: doublet of doublet; s: singlet; n.o.: not observable.

**Table 3:** Validation parameters of analytical method for crocetin quantification (results are expressed in ng eq. *trans*-crocetin/mL of serum).

Linearity and Sensitivity					
Linear Equation	Regression Coefficient (r^2^)	LOD (ng/mL)		LOQ (ng/mL)	
y = 0.241 x − 0.031	0.9988	0.09		0.3	
**Precision and accuracy**					
**Nominal concentration (ng/mL)**	**Concentration found (ng/mL)**	**Precision (%)**		**Accuracy (%)**	
		**Intra-day**	**Inter-day**	**Intra-day**	**Inter-day**
3.0	3.1 +/− 0.2	2.3	5.7	−0.5 to 13.4	6.0
16.0	15.1 +/− 0.6	3.4	4.1	−3.5 to −8.9	−5.6
29.0	27.0 +/− 0.8	2.7	3.1	−5.2 to −10.0	−7.9
**Recovery and matrix effect**					
**Recovery**			**Matrix effect**		
**Nominal concentration (ng/mL)**	**Mean +/− SD**	**RSD**	**Nominal concentration (ng/mL)**	**Mean +/− SD**	**RSD**
3.0	106.0 +/− 6.0	5.7	1.4	93.4 ± 2.6	2.8
16.0	94.4 +/− 3.8	4.1	14.0	96.6 ± 1.1	1.1
29.0	92.1 +/− 2.9	3.1			
**Stability**					
**Conditions**		**Spiked concentration (ng/mL)**	**Measured concentration (ng/mL)**	**Precision** **(RSD %)**	**Accuracy (RE %)**
**Temperature (°C)**	**Time**				
Room Temperature	4 h	14	13.2 ± 0.1	0.5%	−8.5%
4	24 h		14.3 ± 0.8	5.6%	−0.6%
−20	1 month		13.3 ± 0.5	3.6%	−7.3%
3 Freeze–thaw			12.6 ± 0.3	2.0%	−12.4%

## Data Availability

The data presented in this study are available upon request from the corresponding author. The data are not publicly available due to ethical restrictions.
